# Implementation of an Approach to Equitable Allocation of SARS-CoV-2 Monoclonal Antibodies for Preexposure Prophylaxis: Experience From a Single Medical Center

**DOI:** 10.1093/ofid/ofae388

**Published:** 2024-07-10

**Authors:** Keith W Hamilton, Elvis Hua, Lauren Dutcher, Holly Fernandez Lynch, Paul Junker, Abigail G Doucette, Danielle Werner, Ethan Z Kannel, Thomas Civitello, Peter Gabriel, Vivek N Ahya, Dina A Jacobs, Alfred Garfall, Keith Pratz, Kathleen O Degnan, Emily A Blumberg, Donna Capozzi, Ethan Craig, Patricia Takach, Aimee S Payne, Abdallah Geara, Helen Koenig, Lawrence Holzman, Pablo Tebas

**Affiliations:** Division of Infectious Diseases, Perelman School of Medicine at the University of Pennsylvania, Philadelphia, Pennsylvania, USA; Department of Pharmacy, Hospital of the University of Pennsylvania, Philadelphia, Pennsylvania, USA; Division of Infectious Diseases, Perelman School of Medicine at the University of Pennsylvania, Philadelphia, Pennsylvania, USA; Department of Medical Ethics and Health Policy, Perelman School of Medicine at the University of Pennsylvania, Philadelphia, Pennsylvania, USA; Hospital of the University of Pennsylvania, Philadelphia, Pennsylvania, USA; Abramson Cancer Center, Perelman School of Medicine at the University of Pennsylvania, Philadelphia, Pennsylvania, USA; Clinical Practices of the University of Pennsylvania, Philadelphia, Pennsylvania, USA; University of Pennsylvania Health System, Philadelphia, Pennsylvania, USA; University of Pennsylvania Health System, Philadelphia, Pennsylvania, USA; Abramson Cancer Center, Perelman School of Medicine at the University of Pennsylvania, Philadelphia, Pennsylvania, USA; Clinical Practices of the University of Pennsylvania, Philadelphia, Pennsylvania, USA; Division of Pulmonary, Allergy, and Critical Care, Perelman School of Medicine at the University of Pennsylvania, Philadelphia, Pennsylvania, USA; Department of Neurology, Perelman School of Medicine at the University of Pennsylvania, Philadelphia, Pennsylvania, USA; Division of Hematology and Oncology, Perelman School of Medicine at the University of Pennsylvania, Philadelphia, Pennsylvania, USA; Division of Hematology and Oncology, Perelman School of Medicine at the University of Pennsylvania, Philadelphia, Pennsylvania, USA; Division of Infectious Diseases, Perelman School of Medicine at the University of Pennsylvania, Philadelphia, Pennsylvania, USA; Division of Infectious Diseases, Perelman School of Medicine at the University of Pennsylvania, Philadelphia, Pennsylvania, USA; Oncology Pharmacy and Investigational Drug Services, Hospital of the University of Pennsylvania, Philadelphia, Pennsylvania, USA; Division of Rheumatology, Perelman School of Medicine at the University of Pennsylvania, Philadelphia, Pennsylvania, USA; Corporal Michael J. Crescenz VA Medical Center, Philadelphia, Pennsylvania, USA; Section of Allergy and Immunology, Division of Pulmonary, Allergy, and Critical Care, Perelman School of Medicine at the University of Pennsylvania, Philadelphia, Pennsylvania, USA; Department of Dermatology, Vagelos College of Physicians and Surgeons, Columbia University, New York, New York, USA; Division of Renal, Electrolyte, and Hypertension, Perelman School of Medicine at the University of Pennsylvania, Philadelphia, Pennsylvania, USA; Division of Infectious Diseases, Perelman School of Medicine at the University of Pennsylvania, Philadelphia, Pennsylvania, USA; Division of Renal, Electrolyte, and Hypertension, Perelman School of Medicine at the University of Pennsylvania, Philadelphia, Pennsylvania, USA; Division of Infectious Diseases, Perelman School of Medicine at the University of Pennsylvania, Philadelphia, Pennsylvania, USA

**Keywords:** allocation, COVID-19, disparity, preexposure prophylaxis, SARS-CoV-2 monoclonal antibodies

## Abstract

**Background:**

During the COVID-19 pandemic, SARS-CoV-2 monoclonal antibodies for preexposure prophylaxis (SMA-PrEP) offered patients who were immunocompromised another option for protection. However, SMA-PrEP posed administrative, operational, and ethical challenges for health care facilities, resulting in few patients receiving them. Although the first SMA-PrEP medication, tixagevimab and cilgavimab, had its authorization revoked due to compromised in vitro efficacy, new SMA-PrEP medications are currently completing clinical trials. This article provides an operational framework for administrative organization, patient identification and prioritization, equitable medication allocation, medication ordering and administration, and patient tracking.

**Methods:**

A retrospective cohort study evaluating our hospital's SMA-PrEP administration strategy was performed. Multivariable logistic regression was used to examine factors associated with receipt of SMA-PrEP.

**Results:**

Despite the barriers in administering this medication and the scarcity of resources, our hospital was able to administer at least 1 dose of SMA-PrEP to 1359 of 5902 (23.0%) eligible patients. Even with the steps taken to promote equitable allocation, multivariable logistic regression demonstrated that there were still differences by race, ethnicity, and socioeconomic status. As compared with patients who identified as Black, patients who identified as White (odds ratio [OR], 1.85; 95% CI, 1.46–2.33), Asian (OR, 1.59; 95% CI, 1.03–2.46), and Hispanic (OR, 1.53; 95% CI, 1.02–2.44) were more likely to receive SMA-PrEP. When compared with patients with low socioeconomic status, patients with high socioeconomic status (OR, 1.37; 95% CI, 1.05–1.78) were more likely to be allocated SMA-PrEP.

**Conclusions:**

Despite efforts to mitigate health care disparities, differences by race/ethnicity and socioeconomic status still arose in patients receiving SMA-PrEP.

## Teaser

SARS-CoV-2 monoclonal antibodies for preexposure prophylaxis offer promise for reducing risk of COVID-19 infection in patients who are immunocompromised, but they pose many logistical challenges. This article provides an organized framework to approach ethical allocation and administration of these medications.

## Overview

Protection from COVID-19 vaccination is reduced in patients who are immunocompromised [1–8]. On 8 December 2021, the Food and Drug Administration (FDA) granted emergency use authorization (EUA) for monoclonal antibodies tixagevimab and cilgavimab (TGM/CGM) for preexposure prophylaxis, offering another option [9]. Several studies have suggested reduced incidence of symptomatic COVID-19 infection in patients who are immunocompromised when TGM/CGM is used in conjunction with COVID-19 vaccination [10–12].

After the FDA granted EUA for TGM/CGM, health care facilities were left to determine how to administer a medication that posed many logistical barriers, such as eligible patient identification, large-volume intramuscular injection, and prolonged observation period. As a result of these challenges, few eligible patients received doses [13]. The EUA for TGM/CGM was revoked 26 January 2023 due to compromised in vitro neutralizing ability against Omicron variants. However, other SARS-CoV-2 monoclonal antibodies for preexposure prophylaxis (SMA-PrEP) have been authorized, including pemivibart (VYD222), or are completing clinical trials, including sipavibart (AZD3152), offering additional options [14, 15]. Unlike other drugs approved by EUA, though, these drugs will be distributed through commercial channels, creating considerable cost considerations for health care facilities. The main goals of this article are to describe (1) our hospital's considerations and approaches to equitable allocation of TGM/CGM and (2) allocation results of the program that we developed. In so doing, we aim to provide a framework for allocating SMA-PrEP and other medications that pose substantial administrative barriers.

### Scientific Considerations

TGM/CGM and pemivibart were authorized by the FDA through EUA. It is likely that future SMA-PrEP options will be authorized by the same mechanism, at least initially [16]. The EUA designation is not equivalent to FDA approval. When new SMA-PrEP agents are authorized, it is likely that this authorization will be based on in vitro data, such as neutralizing ability against circulating SARS-CoV-2 variants [14, 15]. Unlike when SMA-PrEP first became available, almost every American is now estimated to have antibodies to SARS-CoV-2 from infection, vaccination, or both, markedly reducing hospital admissions and mortality [17]. Although the outcomes of these studies do not include hospitalization or mortality, it would be reasonable to extrapolate clinical benefit for some patients, including those with severe B-cell deficits unlikely to respond to vaccination [18].

Further complicating appraisal of benefits and risks of SMA-PrEP is the rapidly changing SARS-CoV-2 viral epidemiology. The in vitro neutralizing capacity of SMA-PrEP may be significantly different with newer variants as they emerge, which may reduce clinical effectiveness [19].

Due to this complexity, establishing a local expert panel or committee to facilitate decision making can be helpful. This expertise can be formalized in a specific COVID-19 Therapeutics Committee, as previously described in our health care system, or through another existing committee [20]. This committee can apply existing scientific evidence to local settings, balancing it with recommendations of public health agencies, regional data on circulating variants, and operational realities in each facility (eg, physical space and staffing) [21].

### Practical Considerations

The process of administering EUA medications is onerous, often requiring unreimbursed efforts and volunteerism by clinicians. These considerations will be even more salient with pemivibart and sipavibart [14, 15]. Unlike TGM/CGM, current EUA submissions would have these medications initially authorized for intravenous, not intramuscular, administration. The 1-hour infusion time and 2-hour observation period of pemivibart creates substantial challenges [15]. Fortunately, many patients at high risk have contact points with the health care system where intravenous SMA-PrEP may be more feasible to administer (eg, hospitalization for solid organ or bone marrow transplant, scheduled infusion of another medication by outpatient infusion center or home infusion) such that they may not need separate visits for administration; however, the added infusion time and observation period are still a substantial barrier. In the inpatient setting where administration may be the most feasible, the reimbursement structure for medications in hospitalized patients may be a disincentive because hospitals may not get additional payment for using these potentially costly medications [22].

New SMA-PrEP will be supplied commercially, and the cost of the medications will fall to health care facilities, insurance payors, and patients, unlike prior SMA-PrEP. Health care facilities face financial constraints that shape operational capabilities, making it challenging to implement treatments that do not align with economic interests, reimbursement models, staffing capabilities, or physical space capacities. Health care facilities therefore will face the decision of whether the resources and staffing required to administer these medications are justifiable. As such, these additional costs will significantly affect processes and utilization.

Additional data ultimately may allow for intramuscular administration of at least 1 agent [14]. Eventual authorization of SMA-PrEP by intramuscular injection would make administration more feasible. Finding methods to fairly reimburse health care facilities may also support SMA-PrEP administration. Utilizing strategies such the Centers for Medicare and Medicaid Services’ New COVID-19 Treatments Add-On Payments may help [22]. Another approach that can augment the efforts of individual health care facilities is by supporting additional administration sites (eg, federally qualified health centers, other freestanding clinics, and retail pharmacies) to deliver SMA-PrEP, assuming that SMA-PrEP can be administered intramuscularly in the future.

No matter how quickly drug production is scaled up, the initial stages of availability of any drug are faced with supply shortages. Therefore, it is likely that SMA-PrEP administration will be limited by drug availability, administration capacity, or both. Health care facilities must first decide whether the population they serve is one that would have a substantial benefit. For some health care facilities, resources required to administer SMA-PrEP may be better placed elsewhere. If SMA-PrEP is offered, narrower eligibility criteria may be prudent to target it to patients most likely to benefit.

### Bioethical Considerations

Health care facilities that elect to administer SMA-PrEP can create prioritization schema that treats patients equitably and favors individuals who are the most vulnerable [23]. One method to maximize equity is to identify patients at the highest risk and narrowly define eligibility criteria such that the eligible population better matches the available drug supply and administration capacity. These criteria can be defined by a local committee as previously described. An example is shown in Table 1. Eligibility criteria can be liberalized as additional supply, intramuscular administration, and administration capacity become available.

**Table 1.** Sample Criteria for Identifying Patients at Highest Risk for Lack of Adequate SARS-CoV-2 Vaccine Response

Diagnosis or medication eligibility	Specific eligibility criteria
SOT	Lung transplant recipients All SOT patients who received T-cell (ATG, alemtuzumab) or B-cell (rituximab) depletion agents within 1 y All SOT patients with pulse dose steroids within 3 mo All SOT patients within 1 y from transplant All SOT patients taking belatacept
Hematologic malignancy	CAR-T therapy Allo-HSCT within 1 y Auto-HSCT within 6 mo Acute lymphocytic leukemia AML/MDS with venetoclax Lymphoma on therapy within 1 y Anti-CD20/52 medication within 1 y GVHD on immunocompromising medication within 6 mo Receipt of ATG within 1 y Multiple myeloma if anti-CD38/anti-BCMA within 1 y Aplastic anemia
Congenital/acquired immunodeficiency	Common variable immunodeficiency Hypogammaglobulinemia requiring immunoglobulin therapy Agammaglobulinemia—X-linked or autosomal recessive Hyper-IgM syndrome Severe combined immunodeficiency Wiskott-Aldrich syndrome Hyper-IgE syndrome (STAT3 or DOCK8) Any patients with severe-enough immunodeficiency that they require immunoglobulin therapy DiGeorge syndrome (22q deletion syndrome) requiring prophylactic antibiotics HIV with CD4 cell count <50/mm^3^ within past 6 mo
Anti-CD19/20/52, BAFF inhibitor, or S1PR modulator treatment	All patients receiving anti-CD19/20/52, BAFF inhibitor, or s1PR modulator therapy within 1 y Patients with a history of rituximab (even if received >1 y prior) who have persistently impaired humoral immunity and require immunoglobulin therapy
Contraindication to COVID-19 vaccine	Medical contraindication to COVID-19 vaccine without completion of recommended vaccine doses
Vaccine nonresponse eligibility	Any patient with an immunocompromising condition or an immunocompromising medication AND demonstrated nonresponse to COVID-19 vaccine by antibody testing. Nonresponse to COVID-19 vaccine is defined as being up-to-date on vaccine administration and having a negative SARS-CoV-2 receptor-binding domain/spike protein IgG test.

Abbreviations: allo-HSCT, allogeneic hematopoietic stem cell transplant; AML, acute myeloid leukemia; auto-HSCT, autologous hematopoietic stem cell transplant; ATG, antithymocyte globulin; BCMA, B-cell maturation agent; CAR-T, chimeric antigen receptor T cell; GVHD, graft-vs-host disease; MDS, myelodysplastic syndrome; SOT, solid organ transplant.

Even with narrowed eligibility criteria, however, there may be inadequate supply and/or administration capacity. In this case, a lottery system may be considered to promote equity [24, 25]. First-come, first-serve approaches favor those with resources and other privileges: allowing patients to receive treatment on that basis exacerbates existing disparities, including those faced by minoritized racial and ethnic groups and those who face socioeconomic disadvantages [26, 27]. These disparities have resulted in a disproportionate impact from COVID-19 on disadvantaged and minoritized populations [26, 27]. To address these disparities, health care facilities can weight lottery chances more heavily in favor of patients who would otherwise fall behind [24, 25]. When a weighted approach is employed, more patients from disadvantaged populations have been shown to receive SMA-PrEP [24, 28, 29]. Yet, weighted lotteries may impose reverse bias, limit utilization by populations actively seeking treatment, and slow operations; therefore, use of this strategy should be balanced against operational realities.

There are several methods by which socioeconomic status can be integrated into patient prioritization. Interactive mapping functions such as the University of Wisconsin's Neighborhood Atlas can be utilized to determine the Area Deprivation Index (ADI), or decile of relative socioeconomic deprivation, based on patient address [30, 31]. ADIs in the 8th–10th decile can be used to indicate high socioeconomic deprivation, and allocation probabilities can be weighted accordingly [24, 25]. The Social Vulnerability Index (SVI) is an alternative to the ADI that incorporates race and ethnicity alongside socioeconomic indicators [32]. Approaches to allocation that explicitly incorporate race and ethnicity can increase legal risk, but failure to do so can result in less benefit to minoritized groups; this trade-off must be considered when selecting between ADI and SVI [33–35]. Using the University of Wisconsin's Neighborhood Atlas to determine ADI may be practical if the patient population is small enough to manually calculate this measure [30, 31]. However, larger populations require more advanced approaches, as described in the Methods section.

**Figure 1.**
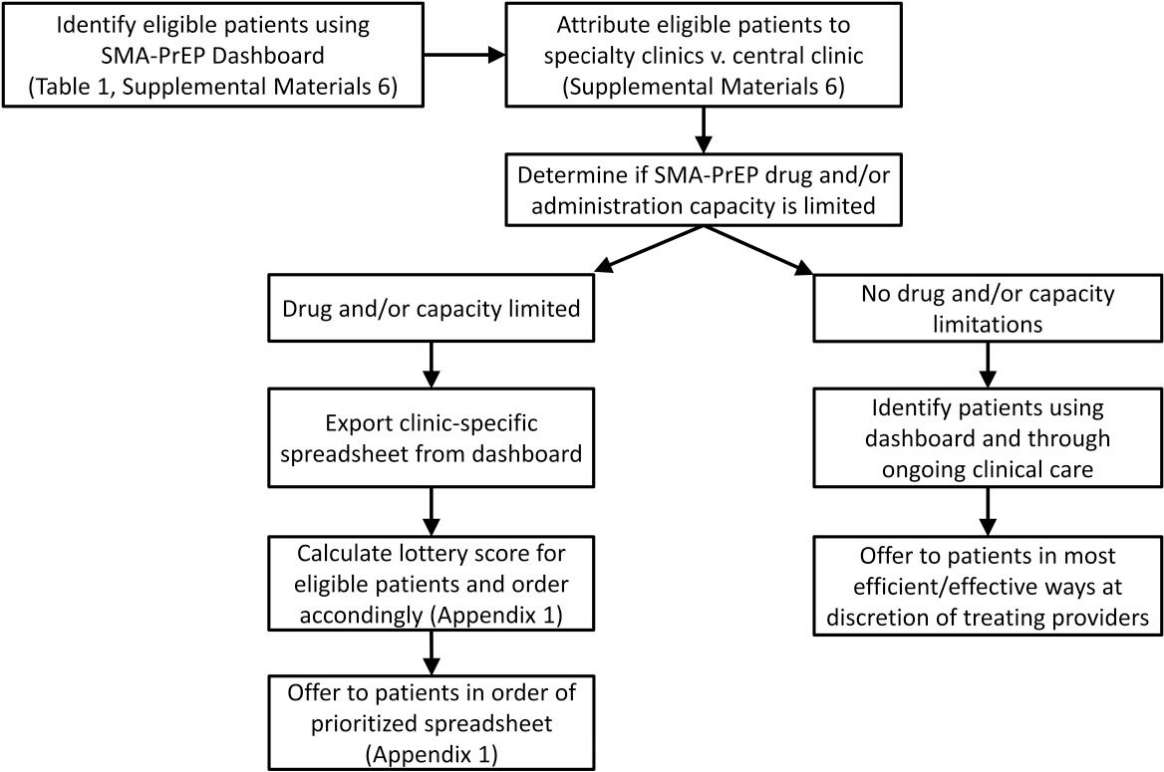
Flow diagram of allocation process for SARS-CoV-2 monoclonal antibodies for preexposure prophylaxis (SMA-PrEP).

A sample lottery protocol is described in Supplementary Material 1 and Figure 1. Even if medication supply and administration capacity are such that performing a lottery is not needed, prospectively identifying patients at high risk and directing outreach to them may reduce disparities.

### Legal and Risk Management Considerations

Because SMA-PrEP medications are authorized by EUA, additional legal and risk management factors should be considered. Unlike investigational new drug applications, EUA medications do not require approval by institutional review boards [36]. However, at a minimum, prescribers must provide patients and/or families with the EUA fact sheet, review the information included therein, and obtain patient or legally authorized representative verbal consent. Finding ways to provide this information effectively and efficiently can be beneficial to prescribers and patients. In many electronic health records (EHRs), this information can be provided through standard messages in messaging portals, with an example provided in Supplementary Material 2. Yet, not every patient has access to EHR portals, so providing information solely via this mechanism is not adequate [37].

Health care facility leaders should review state regulations and discuss with legal counsel because it is possible that added requirements may be placed on prescribing EUA medications in certain locations. Although signed consent is not required by EUA, some institutions may require formal documentation of signed consent. If institutions prefer this approach, a template for signed consent that can be adapted for new EUA medications is presented in Supplementary Material 3, and a template for standard documentation and verbal consent for subsequent dosing is provided in Supplementary Material 4. Even if only verbal consent is obtained, standardizing documentation of the consent discussion is advisable.

Similarly, there may be state regulations or hospital policies regarding which physician and nonphysician health care practitioners can prescribe EUA SMA-PrEP and seek consent. Health care facility leaders planning to administer SMA-PrEP should discuss with legal counsel and credentialing specialists to ensure that administration plans align with regulations and that necessary facility policies and collaborative agreements are in place.

### Identification and Tracking of Eligible Patients

There are several methods of identifying eligible patients, none of which are mutually exclusive: patient self-referral, clinician referral, and central identification. One advantage of patient and/or clinician referral is that there does not need to be an infrastructure to identify eligible patients. Disadvantages of utilizing only referral processes are that patients from disadvantaged groups may be overlooked and patients at highest risk may not receive SMA PrEP. If referral processes are utilized, they could be streamlined by setting up electronic referrals within the EHR to collect relevant information and facilitate screening (Supplementary Material 5).

Supplementing a referral process with proactively identifying eligible patients and performing outreach can facilitate patients at highest risk receiving SMA-PrEP. For some health care facilities small enough to do so, this process may simply involve certain specialties reviewing patient lists and identifying patients at highest risk. Regardless of the approach, specific eligibility criteria for patients at highest risk can be developed to minimize bias.

Given that SMA-PrEP medications have redosing periods, tracking eligible patients can ensure repeat dosing when warranted [15]. Strategies to ensure redosing include (1) writing treatment plans for redosing similar to other health care facility–administered medications requiring repeat dosing (eg, rituximab), (2) developing patient-level automated reminders in the EHR (similar to reminders used for vaccines), and/or (3) building reports or reminders in a SMA-PrEP dashboard to facilitate population health management.

### Distribution Strategies

Following identification of eligible patients, mechanisms of SMA-PrEP ordering and administration need to be established; these mechanisms can be centralized, decentralized, or both. For smaller health care facilities, centralized processes of patient identification, referral, and administration, where specific staff are centralized in 1 administration site, may make the most sense. If the route of administration is intravenous, centralizing the process may be necessary given that there are limited locations where intravenous medications can be administered.

However, for larger health care facilities, the scope and resources of a centralized operation may be infeasible. Based on the prioritization principles noted so far, it may be more feasible to implement an approach that (1) allocates supplies of SMA-PrEP to clinics serving patients at the highest risk, assuming that intramuscular administration is an option, and (2) makes clinics responsible for distributing SMA-PrEP according to institutional guidelines.

An additional advantage of a decentralized approach is that it allows specialists who know the patient to offer SMA-PrEP, which takes out the referral step and may be more effective than having the medication offered by clinicians not known to the patient. Similarly, the process may be more streamlined because SMA-PrEP consenting, ordering, administering, and observation can occur in the context of ongoing clinical care. A disadvantage of exclusively using a decentralized approach is that there may be eligible patients who do not see relevant specialists at the health care facility. Supplementing a decentralized approach with a mechanism to refer these patients to relevant clinics or administration sites may overcome these issues.

## METHODS

The prior sections describe the considerations of the Hospital of the University of Pennsylvania, an academic quaternary medical center, for SMA-PrEP allocation and administration. This section describes the methods of our allocation strategy and for determining allocation outcomes from 24 January 2022 through 25 January 2023. Our health care facility used a combination of decentralized and centralized approaches. We allocated doses of TGM/CGM to clinics that cared for patients at high risk (allergy and immunology, dermatology, hematology-oncology, infectious diseases, nephrology, neurology, rheumatology, and transplant) proportional to the number of patients in each clinic fitting eligibility criteria (Table 1). For centralized administration, we established a central administration site for eligible patients not seen at these clinics who were referred by other clinicians and identified by central clinic staff.

We utilized analytics software (Tableau Software, LLC) to develop a dashboard to identify and track eligible patients. To determine ADI within the dashboard and to weight and prioritize patients with lower socioeconomic status as previously described, we utilized geolocation and block-level socioeconomic stratification. We first generated a list of patients at high risk (Table 1). We then used this list to query the EHR (Epic Systems Corporation), abstracting each patient's current address. We geocoded each address (ArcGIS 10.6.1; ESRI) and transformed addresses into coordinates and US Census Bureau tracts. This census tract was then used to associate each patient with an ADI score [30, 31]. More information on the creation and process of this dashboard can be found in Supplementary Material 6.

The central administration site and specialty clinic leaders were given access to the dashboard, which was used to identify eligible patients and export spreadsheet files that could be used to generate prioritized lists (Supplementary Material 1). Patient outreach was facilitated by electronic communication (Supplementary Material 2), phone calls, and ongoing clinical care. When the supply of TGM/CGM was sufficient such that it was no longer the rate-determining step, patients were offered TGM/CGM without using a lottery process, but the dashboard was still utilized for patient identification and tracking. Based on recommendations from legal counsel, documentation of signed consent for the initial dose (Supplementary Material 3) and verbal consent for subsequent doses (Supplementary Material 4) was required prior to administration.

A retrospective cohort study of eligible patients as defined by the health system's eligibility criteria (Table 1) was performed to determine TGM-CGM administration results of our allocation strategy. Relevant patient diagnoses and demographic characteristics were collected. Socioeconomic status was determined by calculating the ADI according to geolocation and block-level socioeconomic stratification as previously described. For analysis, deciles of socioeconomic status were subcategorized as high (1st–3rd deciles), middle (4th–7th deciles), and low (8th–10th deciles). Descriptive statistics were used to depict the eligible population. Only the first dose of TGM/CGM was evaluated for the purpose of the study.

To determine the association of patient characteristics, such as age, gender, race/ethnicity, and socioeconomic status, with receipt of TGM/CGM, univariable logistic regression analyses were performed on these characteristics as well as relevant diagnoses. Multivariable logistic regression analysis was then performed with variables considered for inclusion if *P* ≤ .20 for univariable analyses. Otherwise, for other analyses, *P* ≤ .05 was considered statistically significant. This study was approved by the Institutional Review Board at the University of Pennsylvania.

## RESULTS

A total of 1359 of 5902 (23.0%) eligible patients received at least 1 dose of TGM/CGM: 148 (10.9%) inpatients and 1211 (89.1%) outpatients. The distribution outcomes of the different clinics administering TGM/CGM are shown in Supplementary Material 7. Patient characteristics and diagnostic groupings for the total eligible population, as well as for patients who received TGM/CGM and for those who did not, are shown in Table 2. Of note, the population that received TGM/CGM differed in many characteristics from the population that did not: age, gender, presence of oncologic qualifying diagnosis and/or treatment, solid organ transplantation, and other immunocompromising condition, as well as receipt of anti-CD19/20/52, BAFF inhibitor, or s1PR modulator medication. Univariable logistic regression yielded comparable results (Table 3).

**Table 2.** Patient Characteristics: Total Eligible Population for TGM/CGM and Patients Who Did and Did Not Receive TGM/CGM

Characteristic	Total Eligible Population (n = 5902)	Received TGM/CGM (n = 1359)	Did Not Receive TGM/CGM (n = 4543)
Age, y	59 (45–69)	64 (53–71)	57 (43–68)
Gender			
Female	3243 (55.0)	650 (47.8)	2593 (42.9)
Male	2659 (45.0)	709 (52.2)	1950 (57.1)
Oncologic diagnosis	2453 (41.6)	645 (47.5)	1808 (39.8)
Solid organ transplant	913 (15.5)	357 (25.3)	556 (12.2)
Heart	78 (1.3)	39 (2.9)	39 (0.9)
Kidney	262 (4.4)	75 (5.5)	187 (4.1)
Liver	113 (1.9)	27 (2.0)	86 (1.9)
Lung	556 (9.4)	250 (18.4)	306 (6.7)
Pancreas	25 (0.4)	9 (0.7)	16 (0.4)
Anti-CD19/20/52, BAFF inhibitor, or s1PR modulator treatment	2531 (42.9)	331 (24.4)	2200 (48.4)
HIV diagnosis	18 (0.3)	1 (0.1)	17 (0.2)
Other immunocompromising diagnosis	34 (0.6)	14 (1.0)	20 (0.4)
ADI national decile			
1%–10%: 1st	721 (12.2)	204 (15.0)	517 (11.4)
11%–20%: 2nd	1142 (19.3)	276 (20.3)	866 (19.1)
21%–30%: 3rd	1094 (18.5)	276 (20.3)	818 (18.0)
31%–40%: 4th	890 (15.1)	213 (15.7)	677 (14.9)
41%–50%: 5th	643 (10.9)	143 (10.5)	500 (11.0)
51%–60%: 6th	435 (7.4)	100 (7.4)	335 (7.4)
61%–70%: 7th	310 (5.3)	55 (4.0)	255 (5.6)
71%–80%: 8th	210 (3.6)	29 (2.1)	181 (4.0)
81%–90%: 9th	211 (3.6)	34 (2.5)	177 (3.9)
91%–100%: 10th	246 (4.2)	29 (2.1)	217 (4.8)
Race/ethnicity			
Asian	162 (2.7)	39 (2.9)	123 (2.7)
Black	925 (15.7)	121 (8.9)	804 (17.7)
Hispanic	155 (2.6)	30 (2.2)	125 (2.8)
More than 1	112 (1.9)	17 (1.3)	95 (2.1)
Other/unknown	346 (5.9)	50 (3.7)	296 (6.5)
White	4202 (71.2)	1102 (81.1)	3100 (68.2)

Data are presented as median (IQR) and No. (%).

Abbreviations: ADI, Area Deprivation Index; TGM/CGM, tixagevimab-cilgavimab.

**Table 3.** Univariable and Multivariable Logistic Regression for Receipt of Tixagevimab-Cilgavimab

	Univariable Logistic Regression			Multivariable Logistic Regression		
Characteristic	Odds Ratio	95% CI	*P* Value	Odds Ratio	95% CI	*P* Value
Age, y						
18–30	1 [Ref]	…	…	1 [Ref]	…	…
31–40	1.68	1.08–2.61	.02	1.76	1.12–2.78	.02
41–50	1.86	1.22–2.86	<.01	1.89	1.21–2.94	<.01
51–60	2.60	1.72–3.94	<.01	2.23	1.45–3.41	<.01
61–70	4.14	2.76–6.20	<.01	2.99	1.96–4.55	<.01
71–80	4.37	2.90–6.58	<.01	2.92	1.90–4.47	<.01
81–90	3.03	1.87–4.91	<.01	2.24	1.35–3.70	<.01
>90	1.86	.59–5.82	.28	1.56	.49–4.95	.45
Female gender	0.69	.61–.78	<.01	0.91	.79–1.03	.13
Oncologic diagnosis	1.36	1.21–1.54	<.01	1.24	1.03–1.43	.03
Solid organ transplant	2.55	2.20–2.97	<.01	1.65	1.34–1.96	<.01
Anti-CD19/20/52, BAFF inhibitor, or s1PR modulator treatment	0.34	.30–.39	<.01	0.08	.05–.15	<.01
HIV diagnosis	0.48	.06–1.18	.22	…	…	…
Other immunocompromising diagnosis	2.35	1.19–4.67	.01	2.12	1.05–4.30	.03
Socioeconomic status: ADI decile						
Low: 8th-10th	1 [Ref]	…	…	1 [Ref]	…	…
Middle: 4th-7th	1.79	1.42–2.28	<.01	1.24	.95–1.61	.11
High: 1st-3rd	2.12	1.69–2.67	<.01	1.37	1.05–1.78	.02
Race/ethnicity						
Black	1 [Ref]	…	…	1 [Ref]	…	…
Asian	2.10	1.40–3.17	<.01	1.59	1.03–2.46	.03
Hispanic	1.59	1.03–2.48	.04	1.53	1.02–2.44	.04
More than 1	1.19	.69–2.06	.54	1.18	.66–2.11	.57
Other/unknown	1.08	.75–1.56	.66	1.15	.78–1.69	.48
White	2.36	1.93–2.89	<.01	1.85	1.46–2.33	<.01

Abbreviations: ADI, area deprivation index; Ref, reference.

After adjustment for patient characteristics and relevant diagnoses, multivariable logistic regression demonstrated that there were still differences by age, race/ethnicity, and socioeconomic status (Table 3). When compared with patients 18 to 30 years old, each subsequent decade of age had increased odds of receiving TGM/CGM until the decade of 71 to 80 years. As compared with patients who identified as Black, patients who identified as White (odds ratio [OR], 1.85; 95% CI, 1.46–2.33), Asian (OR, 1.59; 95% CI, 1.03–2.46), and Hispanic (OR, 1.53; 95% CI, 1.02–2.44) were more likely to receive TGM/CGM. When compared with patients with low socioeconomic status, patients with high socioeconomic status (OR, 1.37; 95% CI, 1.05–1.78) were more likely to receive TGM/CGM.

## DISCUSSION

This article outlines approaches and outcomes of an allocation strategy from 1 health care facility to administer SMA-PrEP. It may provide a blueprint for other health care facilities to define local guidelines, identify patients, create referral and outreach strategies, establish allocation schema, and track outcomes.

Notwithstanding the organized and structured approach taken by our facility, the fact that only 23.0% of eligible patients received TGM/CGM underscores the administration challenges of these medications. The first several months of administration were limited by medication shortage; however, for most of the period in which TGM/CGM was authorized, medication supply was not the limiting factor, and availability was such that all eligible patients could have received doses. Regardless, administration capacity, even with a decentralized approach, limited the speed of administration. Furthermore, several months after authorization, TGM/CGM was noted to have impaired in vitro neutralizing capabilities against circulating Omicron variants, causing the FDA on 24 February 2022 to require an increased dose to overcome the diminished activity [38]. Although specific reasons why patients did not receive TGM/CGM were not prospectively collected, specialty leaders reported a waning interest among patients and clinicians given reports of impaired activity. The perceived onerous process of receiving multiple intramuscular injections in addition to the frequent vaccine boosters may have also resulted in underutilization.

Despite efforts to reduce disparities in administration of TGM/CGM, there were still disparities by race/ethnicity and socioeconomic status. Some reasons why disparities may still have occurred in these patients follow: difficulty in contacting patients; disparate efforts made to encourage patients to receive treatment; barriers to effective communication; disparity in health literacy; decreased trust of clinicians; uninsured or underinsured status; and practical barriers, such as family or work demands, lack of transportation, and disability [39–43]. Community outreach and behavioral economic strategies, as previously described by our institution in the setting of COVID-19 vaccination, could offer promise for further reducing disparities in SMA-PrEP administration [44]. It is also possible that using SVI as opposed to ADI may result in more equity by race and ethnicity, but doing so may increase the risk of legal challenge [34]. Finally, preexisting racial, ethnic, and socioeconomic disparities, which are associated with a higher incidence of chronic disease, may disproportionately affect these groups if health care access, education, and resources are also disparate, limiting the efficacy of even the best-designed allocation framework [45]. Although there were still disparities in administration in this study, health care facilities should still strive to implement measures to mitigate biases to avoid even wider disparities from occurring.

One limitation of this study is that data collection did not quantify reasons why patients did not receive TGM/CGM. Therefore, we are unable to estimate the impact of these factors. Additional studies evaluating outcomes should assess patient motivations and barriers. Another limitation related to the mostly decentralized allocation strategy is that the likelihood of TGM/CGM receipt may have been related to outreach strategies and ease of administration in respective clinics as opposed to diagnoses themselves. Therefore, a causal relationship in differences in allocation based on certain diagnoses should not be inferred because TGM/CGM was allocated in a mostly decentralized fashion at our institution. Nonetheless, these factors were important to control when evaluating the likelihood of receiving TGM/CGM by race/ethnicity, socioeconomic status, age, and gender. In addition, the allocation strategy used only ADI for allocation and analysis purposes, so it is possible that use of another method of determining socioeconomic deprivation (eg, SVI) may have yielded different results. Another limitation of this study is that it discusses the experience from a single medical center. Therefore, the guidance and data may not be applicable to all health care facilities. However, we hope that the experience and data may still offer guidance for other facilities as they plan for administration and allocation of similar medications.

## Supplementary Material

ofae388_Supplementary_Data

## Supplementary Data

Supplementary materials are available at *Open Forum Infectious Diseases* online. Consisting of data provided by the authors to benefit the reader, the posted materials are not copyedited and are the sole responsibility of the authors, so questions or comments should be addressed to the corresponding author.

## Notes

***Acknowledgments.*** We thank Steven Joffe, MD, MPH, for his expertise and input guiding the development of an ethical allocation framework. We also acknowledge the essential role that the Penn Medicine COVID-19 Therapeutics Committee and its members played in the planning and execution of SMA-PrEP administration. Finally, we acknowledge the numerous nurses, pharmacists, clinicians, and personnel involved in frontline administration.

***Patient consent statement***. This study was approved by the Institutional Review Board at the University of Pennsylvania, and a waiver of informed consent was obtained.
