# Supplementary material for: Implementation of an Approach to Equitable Allocation of SARS-CoV-2 Monoclonal Antibodies for Preexposure Prophylaxis: Experience From a Single Medical Center
Source: Open Forum Infect Dis. 2024 Jul 10;11(8):ofae388. doi: 10.1093/ofid/ofae388 (PMC11297503; doi:10.1093/ofid/ofae388)
Supplement: ofae388_Supplementary_Data [file ofae388_supplementary_data.docx]

**Supplemental Materials 1.** Example randomization allocation process for SARS-CoV-2 monoclonal antibodies for pre-exposure prophylaxis (SMA-PrEP) in times of scarcity.

**General Principles**

- Allocation of SMA-PrEP if they are in scarce supply is based off of the framework developed by the Pennsylvania Department of Health: [https://www.health.pa.gov/topics/disease/coronavirus/Pages/Guidance/Ethical- Allocation-Framework.aspx](https://www.health.pa.gov/topics/disease/coronavirus/Pages/Guidance/Ethical-Allocation-Framework.aspx).
- Each specialty clinic will review eligible patients for SMA-PrEP on an ongoing basis throughout business hours (8 am – 5 pm) on business days.
- Doses of SMA-PrEP will be allocated proportionally to facilities and/or specialty clinics who are authorized to order and administer the medication. These allocations from pharmacy will be relative to the proportion of patients that facility and/or specialty clinic makes up of the entire population to which the medication is currently being administered.
- Each clinic will determine the frequency of review based on patient volume and drug availability, and each clinic will make every effort to prospectively identify patients eligible for SMA-PrEP.
- The team or individual at each hospital performing the allocation process should be blinded to information that is not relevant to fair application of the allocation framework, such as patients' names, religion, race, ethnicity, gender, age, sexual orientation, presence of a disability, and immigration status.
- If there is enough SMA-PrEP available at a facility to accommodate all the projected eligible patients until the next resupply in a typical review period and if there is enough capacity to infuse these patients in a given day, then all eligible patients will be offered SMA-PrEP in a process that is most convenient to the administering location or practice.
- If SMA-PrEP supply is scarce and will not accommodate all the projected patients until the next resupply, then an equitable allocation process will be employed on eligible patients to determine which patients will be offered SMA-PrEP.
  - A clinic may be able to use additional objective, pre-determined criteria to identify an extremely high-risk group of patients from within a given patient population to which to administer doses without the need of a lottery if they meet these pre-determined criteria. These criteria should be defined and documented by clinic leadership. Clinics should avoid subjective determination of risk (e.g., asking individual practitioners who they think are highest risk) to avoid bias.
  - If a clinic or specialty defines an extremely high-risk group from within a given population, and there are more than enough doses for this subset of patients, then the remaining doses can be allocated to the remainder of patients within the population using a lottery process. However, if a new patient who fits the extremely high-risk criteria is identified, this patient can be “fast tracked” to receive a dose outside of the lottery process.
  - If a particular group, whether that be an extremely high-risk subset or an entire population of patients, has more patients than eligible to receive the medication, then the medication should be allocated via a lottery process.
- An approach to the lottery process is shown below in the section “Allocation Process When SMA-PrEP Medication Is Scarce.”

**Allocation Process When SMA-PrEP Medication Is Scarce**

1. Determine the number of unassigned doses of SMA-PrEP.
2. Estimate the number of eligible patients in the population for which the SMA-PrEP is currently being administered.
3. Determine the chances for each eligible patient to receive the drug. These chances are determined by dividing the number of available doses (Step 1) by the estimated number of eligible patients (Step 2). For example, if there are 10 doses of drug available and 20 patients expected to be eligible, the “general population” chances to receive the drug are 10 out of 20 (50%).
4. Follow steps to weighted lottery in section “Approach to Weighted Lottery” below.

**Approach to Weighted Lottery**

1. Determine eligibility of each “general population” patient from section above.
2. Determine if the patient has any factors that **augment chances in weighted lottery**:
   1. If possible, determine whether the patient resides in a disadvantaged community, defined as their residential address being in an area with 8^th^, 9^th^, or 10^th^ decile of Area Deprivation Index (ADI).^^[[1]](#footnote-2)^^
   2. If possible, determine if the patient is pregnant.
3. Assign the appropriate weighted score to each patient. Use the following table to assign weight, and enter this number (between 0-1) in the database:

| **Factor** | **Chances to Receive Treatment** |
| --- | --- |
| General community member without any factors that augment chances to receive treatment | Number calculated in “Allocation Process When EUA SMA-PrEP Is Scarce” |
| Community member with factors that augment chances to receive treatment | [1 + (number of factors that augment chances to receive treatment x 0.25)] x (general community chances) |

1. Enter columns for necessary demographic details, general population chances for each patient (see above), pregnancy where 1=pregnant and 0=not pregnant (if possible), and ADI 8-10 where 1=from ADI 8-10 and 0=from ADI not in 8-10 categories (if possible). If pregnancy and/or ADI can be included, create a column that calculates augmented Patient Chances. If these factors cannot be included, then skip this step, and General Population Chances = Patient Chances. If they can be included, add a formula to all cells in the column that calculates the following: General Population Chances * (1 + 0.25 if pregnant + 0.25 if from ADI 8-10).

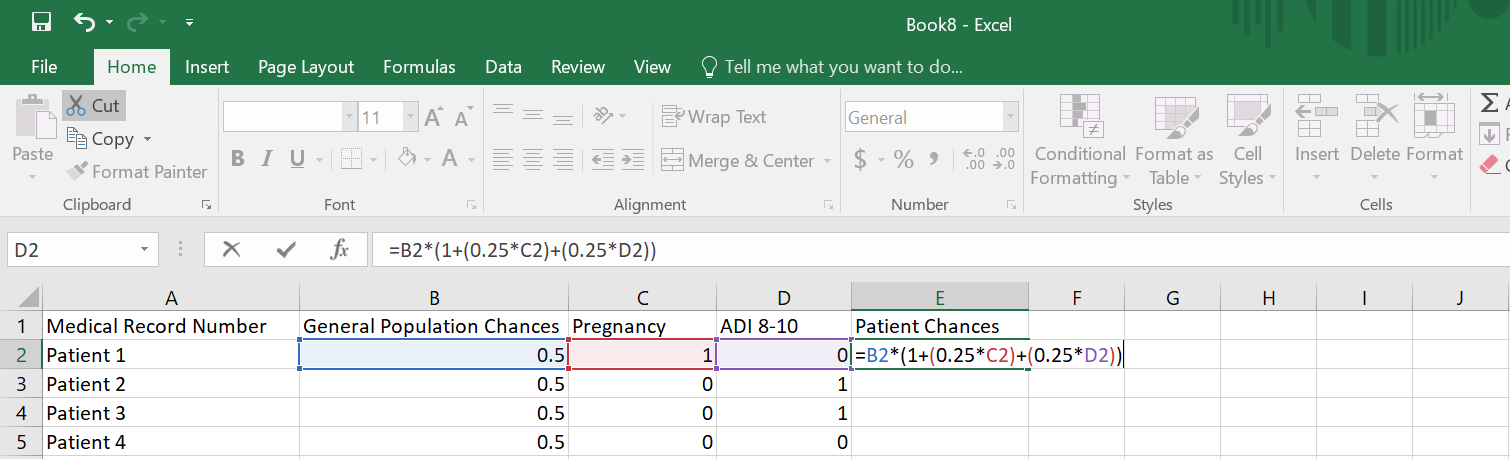

2. Generate a random lottery number (between 0-1) in the “Randomization Process” column for each patient by blocking the column to include all the patient rows as shown and then typing “=RAND().”
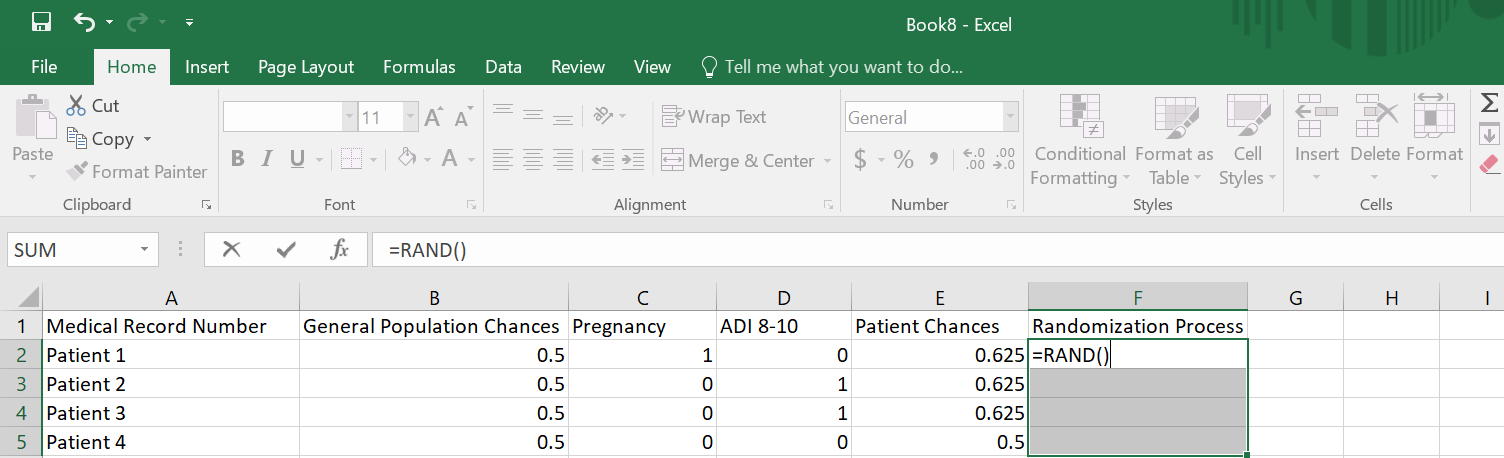

   1. Then press CTRL + Enter, which will generate random numbers from 0 to 1.
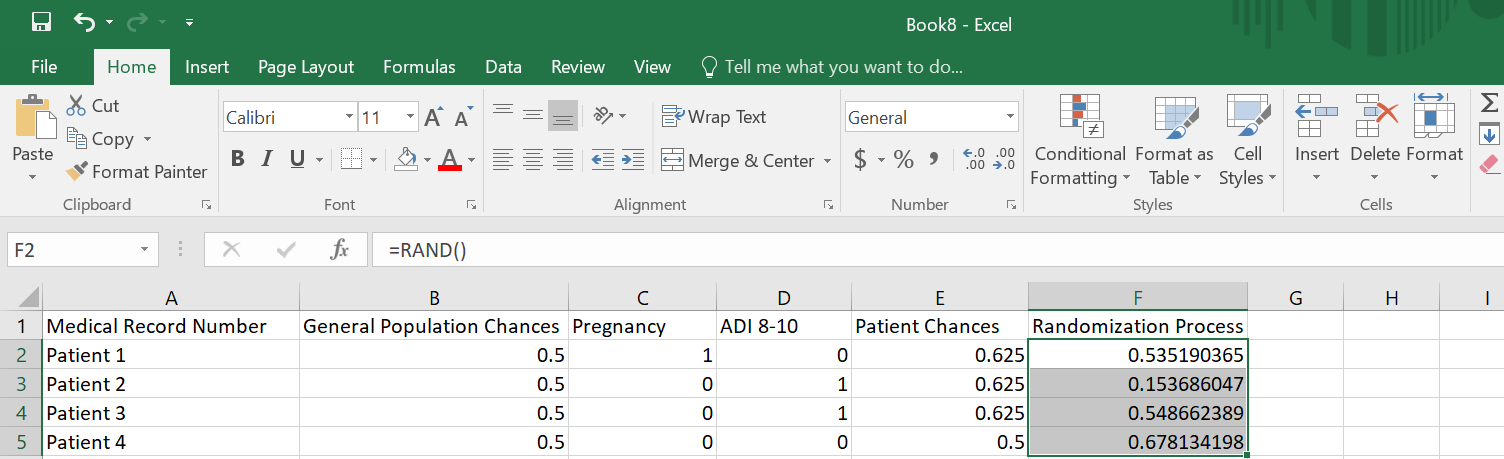

   2. If you do anything to the spreadsheet, these numbers will change; therefore, copy them and paste them into the second column but when you paste, select “Values Only” which will make sure the numbers do not change. It is possible that the numbers will change when you paste them, which is fine. You just need to use these pasted values as the final values for randomization.
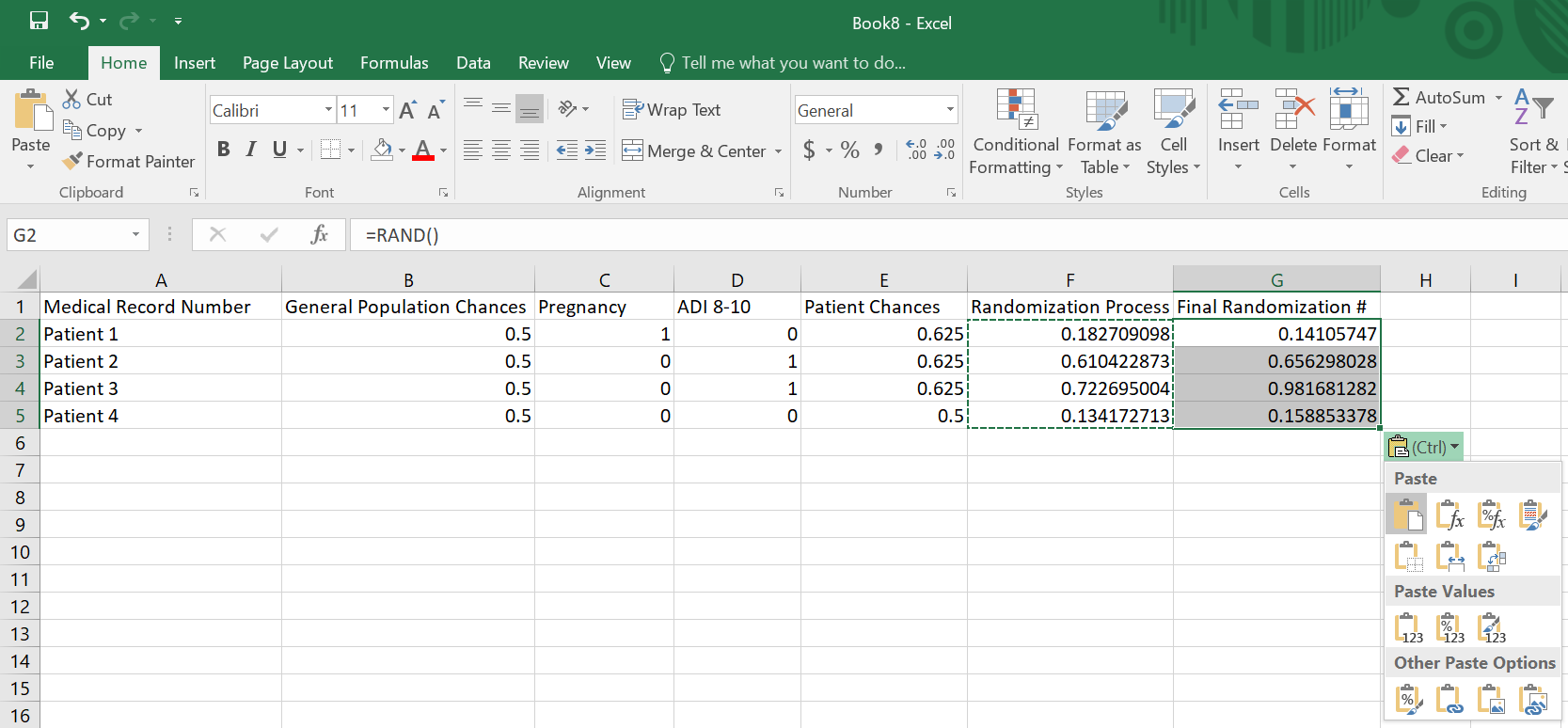

   3. Delete the “Randomization Process” column with the formulas in it, leaving the column with the numeric values only: **these values are the random lottery numbers for each patient**.

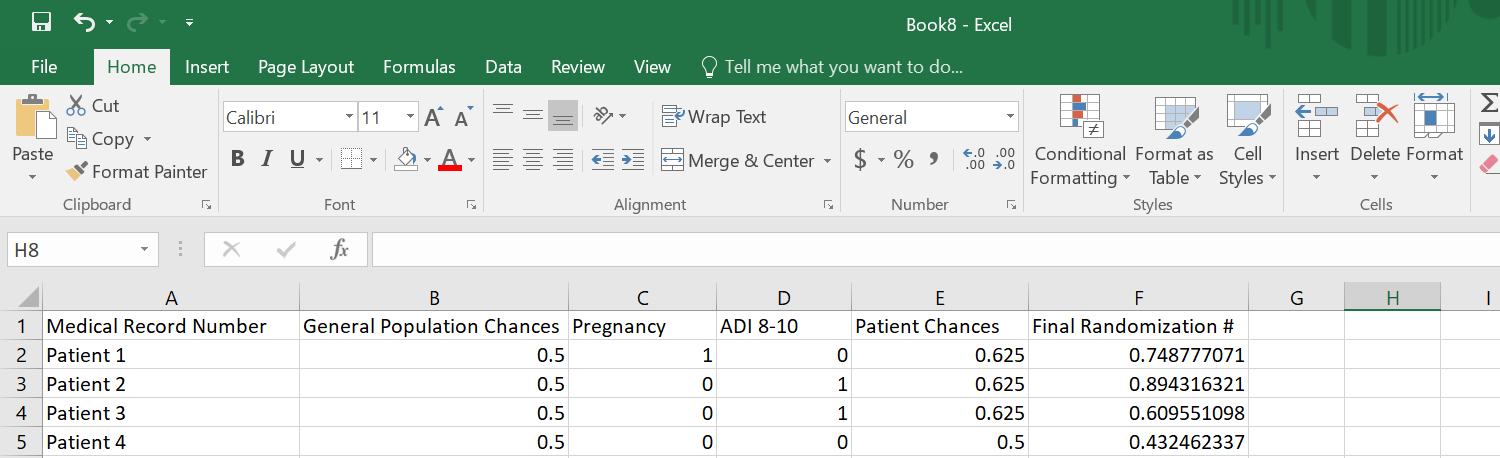

   4. Calculate the sum of the patient chances and the final randomization # by blocking the “Sum of the Chances and Number” column, adding the “Final Randomization #” column to the “Patient Chances” column, and then pressing CTRL + Enter. This sum will now be the random lottery number.

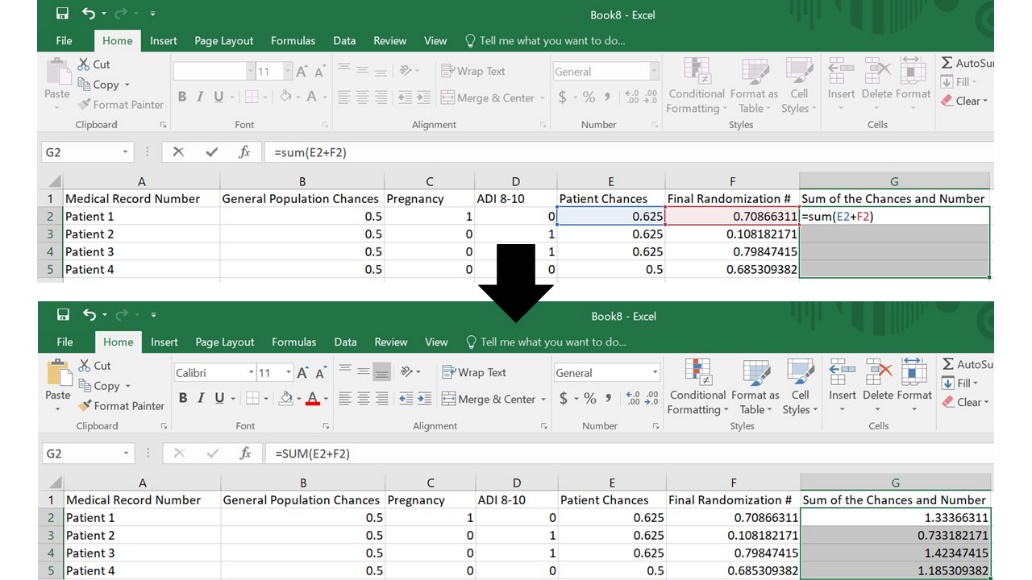

   5. Sort the spreadsheet from largest to smallest by the “Sum of the Chances and Number” column: (1) select that “Data” tab; (2) select the “Sort” icon; (3) make sure the “My data has headers” box is checked; (4) sort by “Sum of the Chances and Number” column; (5) sort from “Largest to Smallest”; and (6) click “OK”. This process will sort the list from the patient who can be contacted first (the first row of data) to the patient who will be contacted last (the last row of data).

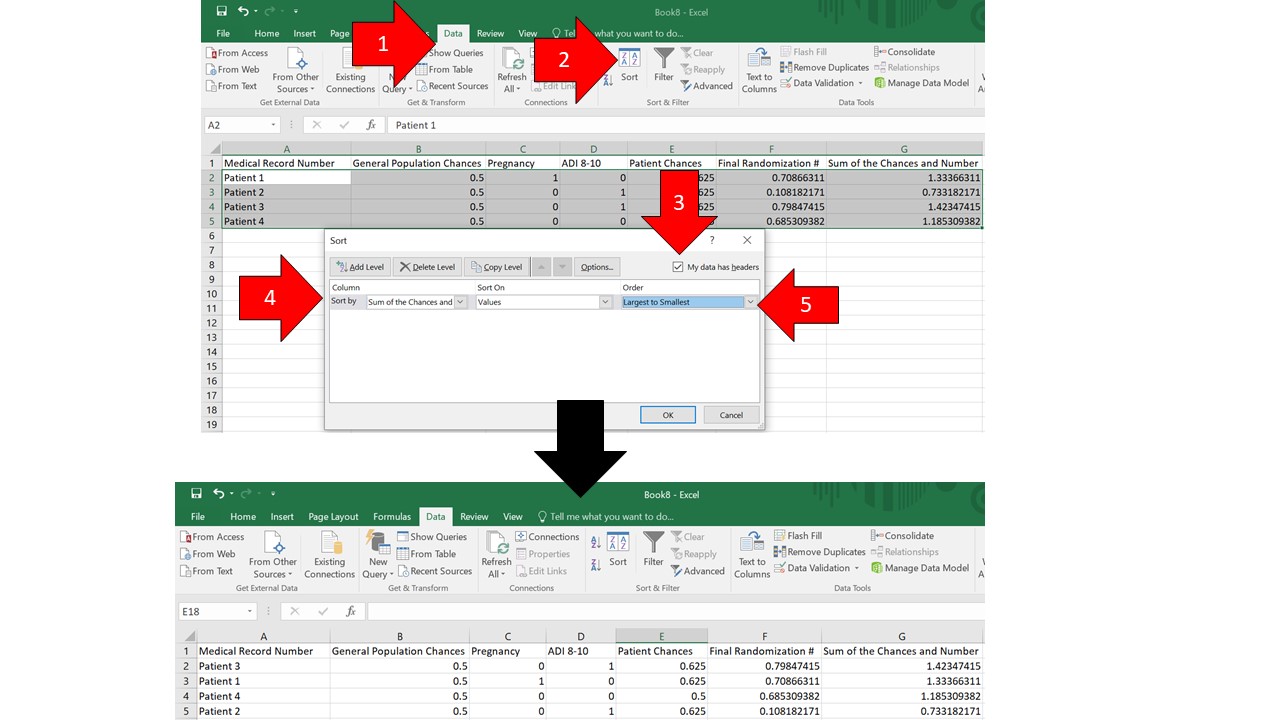

   6. The actual spreadsheets can be saved to a folder on a secure server with a name and date (e.g., “SMA-PrEP_Lottery_Hematology-Oncology_Clinic_12-4-2020”).
3. Determine allocation based on random lottery number. Patients should be offered SMA-PrEP starting from the patient with the highest number to the patient with the lowest number until supply or is exhausted.
4. Because new patients will become eligible or will be referred to receive SMA-PrEP on a regular basis, each clinic should determine how frequently they want to rerun the lottery to allow for new patients to be included. The frequency with which the lottery is rerun should be based on the frequency at which the clinic expects new patients, the rate at which the clinic administers SMA-PrEP, and the clinic workflow. The frequency could be time-based (e.g., once every 2 weeks) or allocation based (e.g., every time a new allocation is received). Ideally, patients who decline to receive SMA-PrEP should be excluded from future lotteries unless they request to be re-added. However, this exclusion may not be possible given the complexity of dealing with large populations of patients. Patients who decline SMA-PrEP cannot be offered the medication within the same lottery allocation, even if they change their mind and want to receive it. Patients who were not selected to receive the medication in a prior lottery allocation can be included in future lotteries. Although doing so increases the chances of any one patient receiving the medication because they will have multiple opportunities to be selected, the expectation is that this medication will become increasingly available so there needs to be a mechanism by which all patients have a chance to receive future allocations even if they have not been selected to receive current allocations.
5. Two members of the team should witness and attest to the correct conduct of the lottery and their names should be recorded on the spreadsheet as witnesses.
6. Patients in the lottery should then be reviewed for eligibility (see “General process for patient identification and eligibility assessment” above).
7. If the patient (or legally authorized representative) is not reached on the first attempt, two additional attempts should be made.

**Audit of Allocation Process**

During times of SMA-PrEP shortage when the allocation process is being utilized, an independent reviewer not involved in the allocation process should audit the process by manually reviewing a random sample of at least 5% of charts (both patients allocated and those not allocated SMA-PrEP) to assess for potential bias in allocation. These reports by the independent reviewers should be signed, dated, and timed by the independent reviewer and stored on a secure server.

Supplemental Materials 2. Standard messaging that can be used for electronic patient outreach or adapted for telephone outreach in patients eligible for SARS-CoV-2 monoclonal antibodies for pre-exposure prophylaxis (SMA-PrEP).

Dear [*PATIENT*],

You may be eligible for a new medication, developed to lower the risk of COVID-19 infection. The medication, [*NAME OF SMA-PREP MEDICATION*], is under **Emergency Use Authorization from the FDA for patients who are moderately to severely immunosuppressed and who have not been recently exposed to or currently have COVID-19.** It has been shown to reduce the likelihood of COVID-19 infection.

The medication is not a substitute for the COVID-19 vaccine, which is still the best defense against COVID-19 infection. [*NAME OF SMA-PREP MEDICATION*] can supplement the COVID vaccine for those who may not have had a full protective reaction.

We invite you to take a brief survey to express your interest and to help us determine whether you are eligible to receive [*NAME OF SMA-PREP MEDICATION*].

**Button or callout: {Take the Questionnaire}**

Learn more about [*NAME OF SMA-PREP MEDICATION*] in the FDA’s [*INSERT LINK TO EMERGENCY USE AUTHORIZATION FACT SHEET*] or contact your care team.

We thank you for trusting us with your care.

Sincerely,

[NAME OF SENDER]

_____________________________________________________

This brief survey will gauge your interest and help determine whether you are eligible to receive [*NAME OF SMA-PREP MEDICATION*] to prevent COVID-19 infection.

1. Are you interested in learning more about this medication?
   1. No – Thank you and have a wonderful day
   2. Yes – Thanks for your interest, we have a series of questions for you to further determine your eligibility – Proceed to next question
2. Have you received [*NAME OF SMA-PREP MEDICATION*] before?
   1. No – Proceed to next question
   2. Yes
      1. If yes, what date did you receive it?
      2. Proceed to next question if scheduling repeat dose at the appropriate administration interval
3. Have you received a COVID-19 vaccine?
   1. No
      1. Why did you not receive a COVID-19 Vaccine? *(default clinical review for patients interested and eligible for SMA-PrEP but have not been vaccinated)*
         1. *I chose not to* – Response to patient: Unfortunately, based on FDA regulations you are not eligible to receive this medication if you have not received a COVID-19 vaccine. Please contact your healthcare practitioner in the future if you decide to receive a COVID-19 vaccine and you are interested in receiving this medication.
         2. My healthcare practitioner recommended that I not receive it or that I wait to receive it.
            1. Proceed to next question
         3. Other:
            1. Proceed to next question
   2. Yes – Proceed to next question
4. Have you received a COVID-19 vaccine in the past 14 days?
   1. If yes what date? Restrict scheduling to at least 14 days after recent vaccine dose, and proceed to next question
   2. No – Proceed to next question
5. Have you been diagnosed with COVID-19, or experienced COVID-19 symptoms in the past 20 days?
   1. Yes
      1. Add note for the scheduler to delay scheduling at least 20 days
      2. Proceed to next question
   2. No – Proceed to next question
6. Have you been exposed to someone with COVID-19 within the past 14 days?
   1. Yes
      1. Add note to the scheduler to delay scheduling for at least 30 days
      2. Proceed to next question
   2. No – Proceed to next question
7. Have you received any monoclonal antibody treatments to treat a COVID-19 infection within the past 3 months?
   1. Yes – Add note for the scheduler to delay scheduling until 3 months after receiving monoclonal antibody treatment
   2. No – Proceed to final messaging

**Final messaging only if patient gets through questionnaire without being disqualified:**

Thank you for answering these questions. Based on your responses you may be eligible to receive [*NAME OF SMA-PREP MEDICATION*]. A team member will contact you to discuss next steps.

Supplemental Materials 3. Example consent template for Emergency Use Authorization (EUA) SARS-CoV-2 monoclonal antibodies for pre-exposure prophylaxis (SMA-PrEP) that can be adapted for other healthcare facilities.

[*HOSPITAL NAME*] Informed Consent Form FOR EMERGENCY USE AUTHORIZATION [*SMA-PREP MEDICATION NAME*]

This consent form is written from the point of view of the patient. If a legally authorized representative will be providing consent, the words “you” and “your” should be substituted for “the patient.”

**What is [*SMA-PREP MEDICATION NAME*]?**

[*SMA-PREP MEDICATION NAME*] is an investigational medication used to prevent Coronavirus Disease 2019 (COVID-19) in patients that may not respond well to COVID-19 vaccines because they have a suppressed immune system. It also can be used in patients who are not able to receive COVID-19 vaccines because of a prior severe reaction to a COVID-19 vaccine or a COVID-19 vaccine component. There is no U.S. Food and Drug Administration (FDA)-approved medication available for this indication. Likewise, [*SMA-PREP MEDICATION NAME*] has not been approved by the FDA. However, the FDA has made this medication available under an emergency access mechanism called an Emergency Use Authorization (EUA). You are receiving this information because your healthcare practitioner believes that this medication may be of benefit to you.

[*SMA-PREP MEDICATION NAME*] is an investigational medication because it is still being studied. It is an antibody that binds to SARS-CoV-2, the virus that causes COVID-19. It may help your immune system to prevent you from getting the infection or making the disease less severe if you were to get it. This document explains the risks and benefits of [*SMA-PREP MEDICATION NAME*].

A healthcare practitioner will go over this document with you. A healthcare practitioner will be available to answer all questions you may have about the information in this document. You also will be given a Fact Sheet for Patients and Parents/Caregivers. We are asking you to read and sign this consent document so that we can be sure you understand the indications for [*SMA-PREP MEDICATION NAME*], its potential benefits, probability of success, likelihood of achieving the desired outcome, recuperative process, potential risks, potential complications, and potential side effects. We also want to be sure that you understand the potential alternatives, including the alternative of refusing this medication, along with the potential risks, complications, and side effects of the alternatives. Please ask questions about anything on this form that you do not understand.

Signing and dating this document also is your acknowledgement that you received a Fact Sheet for Patients and Parents/Caregivers. You will be given a copy of the Fact Sheet and this consent form to keep. It is your choice to receive or not to receive [*SMA-PREP MEDICATION NAME*]. Should you decide not to receive it, it will not affect the other medical care you may receive.

**What is an Emergency Use Authorization (EUA)?**

The FDA has made [*SMA-PREP MEDICATION NAME*] available under an EUA because it has determined that circumstances exist to justify the emergency use during the COVID-19 pandemic. [*SMA-PREP MEDICATION NAME*] has not undergone the same type of review as an FDA-approved or cleared product. The FDA has determined that there are no adequate, approved, or available alternatives to [*SMA-PREP MEDICATION NAME*]. The FDA made its decision on authorizing this medication by EUA based on the totality of scientific evidence available showing that it is reasonable to believe that [*SMA-PREP MEDICATION NAME*] meets certain criteria for safety, performance, and labeling, and that it may be effective.

**What are the benefits of [*SMA-PREP MEDICATION NAME*]?**

[*SMA-PREP MEDICATION NAME*] was shown in clinical trial(s) to [*INSERT EFFICACY DATA FROM CLINICAL TRIAL(S)]*.

**What happens if my healthcare practitioner recommended that I receive [*SMA-PREP MEDICATION NAME*], but I develop symptoms of COVID-19 before I receive it?**

You should talk to your referring healthcare practitioner immediately to obtain a test for COVID-19 and for additional evaluation because [*SMA-PREP MEDICATION NAME*] is not authorized for treatment of individuals who are actively infected with COVID-19.

**What does receiving [*SMA-PREP MEDICATION NAME*] involve?**

[*SMA-PREP MEDICATION NAME*] involves [*DESCRIBE ADMINISTRATION DETAILS* FOR SMA-PREP]. You will be monitored for at least [*LIST LENGTH OF OBSERVATION PERIOD*] after receiving the injections.

**Should I receive additional doses of [*SMA-PREP MEDICATION NAME*] in the future?**

Based on what we know, you will need to receive additional doses of [*SMA-PREP MEDICATION NAME*] every [*INSERT DOSING INTERVAL*] if ongoing protection is needed. Your practitioner will discuss the need for additional doses with you.

**What are the possible risks, complications, and side effects?**

There may be risks involved with taking [*SMA-PREP MEDICATION NAME*], both known and unknown. The specific risks, complications and side effects associated with this medication include but may not be limited to the following:

[*INSERT SIDE EFFECTS AND ESTIMATED FREQUENCY*].

UNKNOWN/UNEXPECTED RISKS AND DISCOMFORTS

There may also be unknown side effects that could harm you because of the medication. We cannot predict what these side effects may be, which is why it is so important for you to report any side effects that you experience to your healthcare practitioner. There is always the possibility that you will have a reaction that, even if treated properly, could be life threatening.

**What things are important to discuss with the practitioner prescribing [*SMA-PREP MEDICATION NAME*]?**

You should discuss all your medical conditions and the medications you are taking with the prescribing practitioner, but specifically you should discuss if you:

- Have any allergies, including if you have had a severe allergic reaction to a COVID-19 vaccine
- Have low numbers of blood platelets (which help blood clotting), a bleeding disorder, or are taking anticoagulants (to prevent blood clots)
- Have had a heart attack or stroke, have other heart problems, or are at high risk for cardiac (heart) events
- Are pregnant or plan to become pregnant
- Are breastfeeding a child
- Have any serious illness
- Are taking any medications (prescription, over-the-counter, vitamins, or herbal products)
- Have received a COVID-19 vaccine dose within the preceding two (2) weeks
- [*INSERT ADDITIONAL CONSIDERATIONS SPECIFIC TO SMA-PREP MEDICATION*]

By signing below, you also are confirming that you have discussed any of these conditions that apply to you with the prescribing practitioner.

**Are there any reasons why I should avoid receiving [*SMA-PREP MEDICATION NAME*]?**

You should avoid receiving [*SMA-PREP MEDICATION NAME*] if you have had an allergy or severe reaction to [*SMA-PREP MEDICATION NAME*] or its components in the past. If you previously experienced a severe reaction to a COVID-19 vaccine, you may be at increased risk for an allergic reaction to [*SMA-PREP MEDICATION NAME*] and may need to consult with an allergist before receiving the medication. [*SMA-PREP MEDICATION NAME*] should be used with caution in individuals with bleeding disorders or on medications that increase the risk of bleeding.

**What are risks of [*SMA-PREP MEDICATION NAME*] if I am pregnant?**

Pregnant women were not included in [*SMA-PREP MEDICATION NAME*] clinical trials so the potential effects of [*SMA-PREP MEDICATION NAME*] on pregnancy and/or an unborn child currently are not known. There are also no data on giving [*SMA-PREP MEDICATION NAME*] to breastfeeding mothers. For a mother and unborn baby, the benefit of receiving [*SMA-PREP MEDICATION NAME*] may be greater than the risk, or it may not be. If you are pregnant or breastfeeding and choose to receive this medication, it is recommended that you:

- Advise your obstetrics practitioner that you have received the medication as well your child’s pediatrician and ensure that your care and that of your child is monitored by a physician
- Talk to your healthcare practitioner about whether you should breast-feed after receiving this medication and the risks that might be involved
- Provide your healthcare practitioner with all the information you know about your health and medications you are taking prior to starting [*SMA-PREP MEDICATION NAME*]
- Tell your healthcare practitioner about any side effects that you may be experiencing after receiving [*SMA-PREP MEDICATION NAME*]

**Is there any problem with receiving [*SMA-PREP MEDICATION NAME*] and a COVID-19 vaccine?**

It is unknown whether [*SMA-PREP MEDICATION NAME*] may reduce the immune response to a COVID-19 vaccine so individuals should wait to receive [*SMA-PREP MEDICATION NAME*] until at least 2 weeks after a COVID-19 vaccination.

**Are there any data on [*SMA-PREP MEDICATION NAME*] and new variants of COVID-19?**

There are limited data on the effectiveness of [*SMA-PREP MEDICATION NAME*] on new COVID-19 variants that were not present at the time [*SMA-PREP MEDICATION NAME*] clinical trial(s) were conducted. It is possible that [*SMA-PREP MEDICATION NAME*] may not be as effective against new variants as was demonstrated in the clinical trials.

**What happens if new information becomes available about this medication?**

Prior to you receiving [*SMA-PREP MEDICATION NAME*], we may receive more information about this medication that could be important to you. This includes information that, once learned, might cause you to change your mind about receiving this medication. We will notify you as soon as possible if such information becomes available.

**What are your other options for COVID-19 prophylaxis?**

At this time, there are no medications that have been approved or authorized by the FDA for this indication. Like [*SMA-PREP MEDICATION NAME*], there may be other medications which have not yet been approved that the FDA has made available for emergency use. There also may be some other experimental medications that your healthcare practitioners may discuss with you as well. If you do develop COVID-19 infection after receiving this medication, you may be eligible to receive other medications used to treat COVID-19.

**What happens if you no longer want to receive [*SMA-PREP MEDICATION NAME*]?**

Your decision to receive [*SMA-PREP MEDICATION NAME*] is voluntary. You can refuse to receive it at any time without giving a reason. There will be no penalty or loss of benefits to you. If you decide not to receive [*SMA-PREP MEDICATION NAME*], it will not affect medical care that you otherwise may receive. A healthcare practitioner may decide for your medical safety to stop administering [*SMA-PREP MEDICATION NAME*] as well. If administration of [*SMA-PREP MEDICATION NAME*] is stopped for any reason, a healthcare practitioner will closely monitor your overall health.

**What is the recuperative process after receiving [*SMA-PREP MEDICATION NAME*]?**

It is not expected that there will be significant recuperative time or processes after receiving [*SMA-PREP MEDICATION NAME*]. However, it is possible that you may have side effects of [*SMA-PREP MEDICATION NAME*] that will require additional treatment.

**How much will [*SMA-PREP MEDICATION NAME*] cost you?**

[*SMA-PREP MEDICATION NAME*] is supplied commercially so there is a cost to the medication, and there may also be a cost of administration. These costs will be billed to your usual health care payor. However, based on your insurance coverage, deductible, and co-pay, you may also need to pay for the medication and/or administration. If you have any questions, about billing, contact [*INSERT CONTACT INFORMATION FOR BILLING OFFICE].*

| When you sign this form, you are agreeing to the following:   - You have carefully read and understand the information in this document. - The purpose and process involved in receiving this medication have been fully explained to you. - You were able to ask questions and all your questions were answered to your satisfaction. - You have been informed that [*SMA-PREP MEDICATION NAME*] is an investigational antibody that is not approved by the FDA but is authorized by the FDA through Emergency Use Authorization to be administered to certain patients to help prevent COVID-19. - You have received a Fact Sheet for Patients and Parents/Caregivers. - There are risks involved with this medication and with associated procedures. You have been informed of the possible risks (including the fact that there may be some unknown and unforeseeable potential risks) associated with receiving [*SMA-PREP MEDICATION NAME*] that could affect you. - You have discussed with the prescribing practitioner any of the conditions listed above that apply to you. - You understand that if continued protection is needed, you may need to receive additional doses of [*SMA-PREP MEDICATION NAME*] every [*INSERT DOSING INTERVAL*]. - You have the option to accept or refuse this medication and are free to withdraw your consent and decide not to receive [*SMA-PREP MEDICATION NAME*] at any time. - The possible effect on your health, if any, of not receiving [*SMA-PREP MEDICATION NAME*], as well as the potential benefits and alternatives to receiving this medication have been explained to you. - You understand deciding not to receive [*SMA-PREP MEDICATION NAME*] will not impact your other medical care and other treatment options. |
| --- |

| **Patient, Legally Authorized Representative, Parent, or Guardian of a Child:** | | |
| --- | --- | --- |
| _____________________________________ | _______________________ | _________ |
| Printed Name and relationship if other than patient | Signature | Date and Time |
|  |  |  |
| **Signature of Attending Physician (or Authorized Professional) prescribing treatment and obtaining consent:** | | |
|  |  |  |
|  |  |  |
| _____________________________________ | _______________________ | _________ |
| Printed Name & Title | Signature | Date and Time |
|  |  |  |
| **Signature of Witness to Patient’s Consent (if applicable):** | | |
|  |  |  |
|  |  |  |
| _____________________________________ | _______________________ | _________ |
| Witness Printed Name | Signature | Date and Time |

Interpreter Identification Number (if services used)

Supplemental Materials 4. Example of standard documentation of verbal consent for administration of subsequent doses of emergency use authorization (EUA) SARS-CoV-2 monoclonal antibodies for pre-exposure prophylaxis (SMA-PrEP).

**Confirmation of Eligibility**:

[*PATIENT NAME*] was previously consented for and received at least one prior dose of [*SMA-PREP MEDICATION NAME*]. I confirm that [*PATIENT NAME*] fits eligibility criteria for receiving a repeat dose of [*SMA-PREP MEDICATION NAME*] and that at least [*INSERT DOSING INTERVAL*] will have elapsed between the prior dose and the timing of the next planned dose.

**Patient and/or Family Discussion and Informed Consent**:

I confirm that the [*pulldown menu: patient; legally authorized representative (insert name): ****] has been provided with and that I have reviewed the current updated [*SMA-PREP MEDICATION NAME*] Fact Sheet for Patients, Parents, and Caregivers: [*INSERT LINK OF EUA FACT SHEET*].

If the patient has experienced a severe reaction to a COVID vaccine, I have discussed that the patient may be at increased risk of an allergic reaction to [*SMA-PREP MEDICATION NAME*] and that the patient may need to consult with an allergist before receiving [*SMA-PREP MEDICATION NAME*].

I confirm that I have discussed with the [*pulldown menu: patient; legally authorized representative (insert name): ****] that, based on what we know about current SARS-CoV-2 variants, the patient will need additional doses of [*SMA-PREP MEDICATION NAME*] every [*INSERT DOSING INTERVAL*] if ongoing protection is needed.

**Side Effects**

I also confirm that I discussed with the [*pulldown menu: patient; legally authorized representative (insert name): ****] that [*INSERT DISCUSSION OF SIDE EFFECTS*].

**Risk Assessment and Risk-Benefit Discussion**

I confirm that I have reviewed this patient’s history and discussed relevant pertinent allergies, bleeding disorders, serious illnesses, heart disease, and pregnancy, and if any of these conditions are present, I have discussed any risks and benefits of receiving [*SMA-PREP MEDICATION NAME*] in relationship to these conditions.

Since the patient last received a dose of [*SMA-PREP MEDICATION NAME*], the patient has the following new conditions that may impact the risks and benefits of receiving [*SMA-PREP MEDICATION NAME*]: [*pulldown menu: the patient has no new relevant conditions; the patient has the following new condition(s): ****]. I have discussed any risks and benefits of receiving [*SMA-PREP MEDICATION NAME*] in relationship to any such new conditions.

All questions were answered and the [*pulldown menu: patient; legally authorized representative (insert name): ****] agreed to the patient receiving [*SMA-PREP MEDICATION NAME*].

[*INSERT ELECTRONIC SIGNATURE AND TIMESTAMP OF CONSENTING PRACTITIONER*]

Supplemental Materials 5. Example of referral order in electronic health record for SARS-CoV-2 monoclonal antibodies for pre-exposure prophylaxis (SMA-PrEP).

**Introduction**

This consult is being placed to refer an outpatient for consideration to receive a SARS-CoV-2 monoclonal antibody (SMA) for PRE-EXPOSURE PROPHYLAXIS (PrEP) OF COVID-19. If the patient is actively being seen in any of the following clinics, then contact the patient’s practitioner in that clinic to ensure the patient is included in that clinic’s allocation strategy and do not submit this order: [*INSERT NAMES OF CLINICS ADMINISTERING SMA-PREP*].

Submitting this order does NOT guarantee that a patient will receive SMA-PrEP. Due to limited resources, patients will be allocated SMA-PrEP via an equitable allocation mechanism based on patient eligibility. If the patient is selected to receive SMA-PrEP, the patient will be notified. If the patient cannot be reached, the patient’s ability to receive SMA-PrEP may be forfeited.

**PATIENTS WITH INCOMPLETE INFORMATION IN THIS REFERRAL FORM WILL NOT BE ELIGIBLE TO RECEIVE SMA-PrEP**

**Eligibility Criteria**

[*INSERT ELIGIBILITY CRITERIA*]

**Branching Logic Questions**

- Q1. Is the patient up to date on COVID-19 vaccination? YES/NO
- (If YES to Q1) Q2. Does the patient have any EXCLUSION criteria? YES/NO
- (If NO to Q2) Q3. Does the patient have any INCLUSION criteria SMA-PrEP? YES/NO
- (If YES to Q3) Q4. Please list all the patient’s INCLUSION criteria: [mandatory text box]
  - Allow to submit order
- (If NO to Q1) Q5. Is the COVID-19 vaccine medically contraindicated in this patient? YES/NO
  - If NO to Q5 > Do not allow to submit order and display: “This patient is not eligible because SMA-PrEP is available only for immunosuppressed patients who are up to date on the COVID-19 vaccine or for patients in which a COVID-19 vaccine is medically contraindicated.”
- (If YES to Q5) Q6. Please explain the reason(s) why the COVID-19 vaccine is medically contraindicated: [mandatory text box]
  - Allow to submit order
- (If YES to Q5) Q7. Does the patient have any criteria that would put the patient at high risk for complications of COVID-19? YES/NO
- (If YES to Q7) Q8. Please list the criteria that put this patient in the highest risk tier of eligibility: [mandatory text box]
  - Allow to submit order

Supplemental Materials 6. Example criteria for development of SMA-PrEP identification and tracking dashboard.

**General Overview**

We utilized analytics software (Tableau Software, LLC, Seattle, WA) to develop an interactive dashboard to identify and track patients by extracting data from the electronic health record (EHR) in the University of Pennsylvania Health System (Epic Systems Corporation, Verona, WI)

**Identification of Eligible Patients**

See Table 1 in the primary publication for an overview of the eligibility criteria for patients. Dashboard criteria in this section correspond to the column categories of the referenced table.

*Hematology-Oncology*

Patients fit hematology-oncology eligibility if the patient fit both of the following criteria:

1. Cancer diagnosis code within the last year in the EHR; a primary cancer diagnosis was assigned as the one appearing most often in the past one year

**AND**

1. Patients from the base cancer cohort who meet any of the following criteria in the past one year:
   1. Received any of the following treatments regardless of diagnosis:
      1. Chimeric antigen receptor T-cell (CAR-T) therapy
      2. Allogeneic hematopoietic stem cell transplantation within one year
      3. Autologous hematopoietic stem cell transplantation within six months
      4. Anti-thymocyte globulin within one year
      5. Blinatumomab
      6. Venetoclax
      7. Anti-CD20 agent (e.g., rituximab) within one year
      8. Anti-CD52 agent (e.g., alemtuzumab) within one year
   2. Primary diagnosis of multiple myeloma and received either of the following:
      1. Anti-B-cell maturation agent (e.g., belantamab, idecabtagene vicleucel, ciltacabtagene autoleucel, elranatamab, teclistamab) within one year **OR**
      2. Anti-CD38 therapy (e.g., daratumumab, isatuximab) within one year
   3. Primary diagnosis of lymphoma and receiving immunosuppressive treatment (e.g., sirolimus, temsirolimus, tacrolimus, cyclosporine) and does NOT have a history of solid organ transplant
   4. Primary diagnoisis of acute lymphocytic leukemia
   5. Primary diagnosis of aplastic anemia
   6. Graft-versus-host disease on immunosuppressive medication within six months
2. Patients who have had SARS-CoV-2 receptor binding domain IgG antibody testing and most recent result is negative

*Solid Organ Transplantation*

The EHR used to develop this dashboard included discrete fields on whether a patient had underwent a solid organ transplantation and which organ(s) were transplanted. Patients were eligible by solid organ transplantation criteria if the patient had:

- - - 1. Received a solid organ transplantation, **AND**
      2. Attended an office visit in transplant clinic within the prior two years, **AND**
      3. Had any one of the following criteria:
         1. Received lung transplant (any time in the past),
         2. Were transplanted in last year (any organ)
         3. Currently receiving belatacept,
         4. Received pulse-dosed steroids within last 6 months,
         5. Received T-cell (e.g., antithymocyte globulin, alemtuzumab) or B-cell (e.g., rituximab) depleting agents within the past one year, **OR**
         6. Had SARS-CoV-2 receptor binding domain IgG antibody testing and most recent result is negative

*Congenital or Acquired Immunodeficiency and Immunosuppressive Therapy*

Patients fit eligibility for congenital or acquired immunodeficiency and immunosuppressive therapy if they had any of the following criteria:

1. Diagnosis code for common variable immunodeficiency
2. Diagnosis code for hypogammaglobulinemia with an active order for immunoglobulin therapy*
3. Diagnosis code for agammaglobulinemia - X-linked or autosomal recessive
4. Diagnosis code for hyper-IgM syndrome
5. Diagnosis code for severe combined immunodeficiency
6. Diagnosis code for Wiskott-Aldrich syndrome
7. Diagnosis code for hyper-IgE syndrome (STAT3 or DOCK8)
8. Any patients with severe enough immunodeficiency that they require immunoglobulin therapy*
9. Diagnosis code for DiGeorge syndrome (22q deletion syndrome) requiring prophylactic antibiotics
10. Diagnosis code for HIV with CD4 cell count <50/mm^3^ within past six months

*** Examples of immunoglobulin therapy: immunoglobulin intravenous, immunoglobulin subcutaneous, Asceniv, Flebogamma, Gammagard, Gammaplex, Octagam, Panzyga, Privigen, Gammaked, Gamunex-C, Cutaquig, Cuvitru, Hizentra, HyQvia

*Anti-CD19, Anti-CD20, Anti-CD52, BAFF inhibitor, or s1PR Modulator Treatment*

Patients fit eligibility for anti-CD20, anti-CD52, BAFF inhibitor, or s1PR modulator treatment if they had any of the following criteria:

1. Received anti-CD19 medication within one year (e.g., inebilizumab)
2. Received anti-CD20 medication within one year (e.g., rituximab, ibritumomab, ocrelizumab, ofatumumab, obinutuzumab, tositumomab, veltuzumab)
3. Received anti-CD52 medication within one year (e.g., alemtuzumab)
4. Received BAFF inhibitor medication within on eyear (e.g., belimumab)
5. Received rituximab (even if received greater than one year prior) but have order for immunoglobulin therapy
6. Received s1PR modulators (e.g., fingolimod, siponimod, ozanimod, ponesimod) within one year prior

*Other Immunosuppressive Disorder or Medication with Negative SARS-CoV-2 Antibody*

Patient had SARS-CoV-2 receptor binding domain IgG antibody testing and most recent result is negative

**Identification and Attribution of Eligible Patients for Specialty Clinics**

The list of clinics below is a hierarchy of patient attribution. The hierarchy was based off the administration capacity of each of the clinic sites with the greater capacity being higher on the list. If a patient met the eligibility criteria above, they were attributed to a clinic if they attended an office visit at that clinic within the prior one year. If they did not fit attribution criteria to a specialty clinic, they were attributed to the central administration site as described in the section below. If the patient was seen at more than one specialty clinic, then the patient was attributed only to the clinic highest on the list in which the patient fit attribution criteria. This attribution was important for equitable allocation when medication supply was limited so that certain patients did not appear on more than one list. Similarly, it helped to reduce duplicate efforts and confusion from multiple clinics reaching out to the same patient.

- - - 1. Hematology-oncology – patients attributed only if they fit hematology-oncology criteria above
      2. Transplant – patients attributed only if they fit solid organ transplant criteria above
      3. Infectious diseases
      4. Neurology – patients attributed only if the patient had qualified due to receiving immunosuppressive medication and that medication was prescribed by neurologist
      5. Rheumatology – patients attributed only if the patient had qualified due to receiving immunosuppressive medication and that medication was prescribed by rheumatologist
      6. Allergy and immunology
      7. Nephrology – patients attributed only if the patient had qualified due to receiving immunosuppressive medication and that medication was prescribed by nephrologist
      8. Dermatology – patients attributed only if the patient had qualified due to receiving immunosuppressive medication and that medication was prescribed by dermatologist

**Identification and Attribution of Eligible Patients Not Attributed to Specialty Clinics**

If a patient fit eligibility criteria for SMA-PrEP but could not be allocated to a specialty clinic by the attribution criteria above, then the patient was attributed to the central administration clinic, which was established in the infectious diseases clinic.

**Integrating Socioeconomic Deprivation Metric**:

To determine area deprivation index (ADI) within the dashboard and to weight in favor of patients with lower socioeconomic status as previously described, we utilized geolocation and block-level socioeconomic stratification. We first generated a list of eligible patients as discussed above. We then used that list of patients to query the EHR and to abstract each patient’s current address. Using their documented street address, city, state, and zip code, we then geocoded each address (ESRI 2022, ArcGIS 10.6.1, Redlands, CA) and transformed the address into coordinates. This geolocator allowed different tiers of accuracy for patient address matching. Most of the time, patient addresses mapped perfectly to a given coordinate. In cases of imperfect matches (e.g., post office address), ArcGIS geolocates to the centroid of the smallest available geographic unit in the provided address. In the case of a post office box, the resulting coordinate is the centroid of the zip code provided. Then, using the most updated data from the US Census Bureau, we attributed a Census Tract to each geolocated address. This attributed Census Tract was then used to associate each patient with an Area Deprivation Index (ADI) score^1-2^.

**Eligibility Review and Patient Denial**:

Prior to offering SMA-PrEP to a patient, a healthcare practitioner in the relevant clinic reviewed the patient to confirm eligibility. If a patient was not eligible for SMA-PrEP, we developed standard documentation with discrete data elements that could be entered in a note in the patient’s chart to confirm review and ineligibility. These discrete data elements were then prospectively extracted by the SMA-PrEP dashboard to flag the patient as ineligible.

If the patient was offered SMA-PrEP, we developed standard documentation with discrete data elements that could be entered in the patient's chart to document that the patient was offered SMA-PrEP but declined. Similarly, if a patient was still considering receiving SMA-PrEP, the corresponding discrete data element could be selected. These discrete data elements were then prospectively extracted by the SMA-PrEP dashboard to flag the patient with the appropriate status.

However, these discrete data elements were variably utilized by clinicians so meaningful data on proportions of patients falling into each of these categories could not be generated.

**References**:

1. Kind AJH, Buckingham W. Making Neighborhood Disadvantage Metrics Accessible: the Neighborhood Atlas. New England Journal of Medicine, 2018. 378: 2456-2458. DOI: 10.1056/NEJMp1802313. PMCID: PMC6051533.
2. University of Wisconsin School of Medicine Public Health. 2020 Area Deprivation Index v2.0. Downloaded from https://www.neighborhoodatlas.medicine.wisc.edu/ January 13, 2021.

**Supplemental Materials 7**. Distribution outcomes for tixagevimab-cilgavimab among clinics administering the medication with eligible patients representing the number of eligible patients mapped to that clinic.

| **Clinic** | **Eligible Patients** | **Administered Patients** | **Percent of Eligible Patients Administered** |
| --- | --- | --- | --- |
| Allergy-Immunology | 7 | 1 | 14.3% |
| Central Site | 1185 | 129 | 10.9% |
| Dermatology | 33 | 4 | 12.1% |
| Hematology-Oncology | 2067 | 635 | 30.7% |
| Infectious Disease | 53 | 8 | 15.1% |
| Nephrology | 98 | 19 | 19.4% |
| Neurology | 1418 | 167 | 11.8% |
| Rheumatology | 183 | 48 | 26.2% |
| Transplant | 858 | 348 | 40.6% |
| **All Clinics Combined** | **5902** | **1359** | **23.0%** |

1. <https://www.neighborhoodatlas.medicine.wisc.edu/mapping> [↑](#footnote-ref-2)
